# Clinical Outcomes of Intramedullary Kirschner Wire Fixation for Unstable Radius and Ulna Fractures in Children: A Prospective Study in Yemen

**DOI:** 10.7759/cureus.78824

**Published:** 2025-02-10

**Authors:** Abdullah Al-Moaish, Anwar Mughalles, Ali M Alhamzi, Bassam T Alqadasi, Haitham M Jowah

**Affiliations:** 1 Department of Surgery, Sana'a University, Sana'a, YEM; 2 Department of Orthopedic Surgery, Al-Thawra Modern General Hospital, Sana'a, YEM; 3 Department of Surgery, Al-Thawra Modern General Hospital, Sana'a, YEM

**Keywords:** clinical outcomes, complications, intramedullary fixation, kirchner wire, minimally invasive surgery, pediatric forearm fractures, resource-limited settings, unstable fractures

## Abstract

Background: Forearm fractures are among the most common pediatric injuries, and unstable fractures often require surgical intervention. Intramedullary Kirchner wire (K-wire) fixation has emerged as a minimally invasive treatment option, offering advantages such as reduced soft tissue damage and shorter hospital stays. This study evaluated the clinical outcomes of K-wire fixation for unstable radius and ulna fractures in children.

Methods: This prospective observational study was conducted at the Al-Thawra Modern General Hospital, Yemen, between January 1, 2019, and February 28, 2020. The study included 23 pediatric patients (aged 5-15 years) with unstable forearm fractures who had failed closed reduction. All patients underwent intramedullary K-wire fixation under general anesthesia. Functional outcomes were assessed using the Price criteria, and complications were recorded. Data were analyzed using SPSS Statistics for Windows, Version 16 (Released 2007; SPSS Inc., Chicago, United States).

Results: The mean age of the patients was 10.4 years (range: 5-15 years), with a male predominance (69.6%, n=16). Road traffic accidents (52.2%, n=12) and falls (47.8%, n=11) were the most common mechanisms of injury. Transverse fractures accounted for 56.5% (n=13) of the cases, followed by oblique fractures (39.1%, n=9). Excellent functional outcomes were achieved in 60.9% (n=14) of the patients, and good outcomes were observed in 30.4% (n=7). Complications included pin site irritation (34.8%, n=8) and superficial wound infection (17.4%, n=4), all of which were resolved with appropriate treatment. The majority of patients (52.2%, n=12) underwent K-wire removal between 12 and 14 weeks postoperatively.

Conclusion: Intramedullary K-wire fixation is a safe and effective treatment for unstable radial and ulnar fractures in children, with excellent functional outcomes and manageable complications. The proposed technique is particularly suitable for resource-limited settings because of its simplicity and cost-effectiveness. Future studies should explore long-term outcomes and compare K-wire fixation with other surgical techniques.

## Introduction

Forearm fractures are among the most common orthopedic injuries in children, accounting for approximately 13-15% of all pediatric fractures [1,2]. These injuries often result from falls or direct trauma, with a bimodal age distribution: boys typically experience peaks at approximately 9 and 13-14 years, while girls show a single peak at approximately 5-6 years of age [3]. Due to the complex anatomy and functional importance of the forearm, fractures in this region can lead to significant complications, including malunion, restricted range of motion, and long-term disability if not managed appropriately [4].

In pediatric patients, most forearm fractures can be successfully treated with conservative methods, such as closed reduction and casting, because of the rapid healing and remodeling potential of children’s bones [5]. However, a small subset of fractures (3-4%) are unstable or irreducible, necessitating surgical intervention to achieve satisfactory outcomes [6]. Traditional treatment options for unstable fractures include closed remanipulation under anesthesia, open reduction and internal fixation (ORIF) with plates, and intramedullary fixation using Kirschner wires (K-wires) [7,8].

Among these options, intramedullary K-wire fixation has gained popularity as a minimally invasive technique that offers several advantages, including reduced soft tissue damage, shorter hospital stays, and improved cosmetic outcomes [9]. Unlike plate fixation, which requires extensive dissection and is associated with a higher risk of complications such as stiffness and scarring, K-wire fixation preserves the periosteal blood supply and allows for early mobilization [10]. Despite these benefits, the clinical outcomes of K-wire fixation for unstable forearm fractures in children remain poorly studied, particularly in resource-limited settings.

This study aimed to evaluate the clinical outcomes of intramedullary K-wire fixation for unstable radius and ulna fractures in children, with a focus on functional recovery, complication rates, and overall patient satisfaction. By providing evidence-based insights into the efficacy of this technique, we hope to contribute to the growing body of literature on pediatric fracture management and to inform clinical decision-making in similar contexts.

## Materials and methods

Study design

This prospective observational study was conducted at Al-Thawra Modern General Hospital, a referral trauma center in Yemen, between January 1, 2019, and February 28, 2020. The study was approved by the Institutional Review Board (IRB) of Al-Thawra Modern General Hospital, and written informed consent was obtained from the parents or guardians of all participants.

Study population

The study included pediatric patients aged 5-15 years with unstable diaphyseal fractures of both the radius and ulna. Unstable fractures were defined as those with unacceptable displacement (>10° of angulation or >50% displacement of the fracture fragments on anteroposterior or lateral radiographs) or failed closed reduction (inability to achieve acceptable alignment after two attempts under anesthesia) [11]. Patients were screened based on clinical and radiographic findings, and those meeting the inclusion criteria were enrolled. The exclusion criteria were open fractures, pathological fractures, refractures, malunited fractures, and age >15 years.

Preoperative preparation

Upon admission, all patients underwent a thorough clinical evaluation, including the assessment of airway, breathing, and circulation (ABC). A complete physical was examined to rule out any associated injuries. Anteroposterior and lateral radiographs of the affected forearm, including the elbow and wrist joints, were obtained to confirm the diagnosis and assess the fracture pattern. Routine blood tests were performed, and patients were prepared for surgery once they were deemed medically stable. Prophylactic intravenous antibiotics (ceftriaxone 50 mg/kg) were administered 30 minutes before the skin incision to reduce the risk of surgical site infection.

Surgical technique

All procedures were performed under general anesthesia while the patient was in the supine position. The affected arm was draped, and the shoulder was abducted to 90°. Fluoroscopy was used to obtain real-time anteroposterior and lateral views of the forearm during the procedure. K-wires of 2.0-3.0 mm diameter were selected based on preoperative radiographic measurements of the medullary canal, ensuring that the wire diameter was approximately 0.5 mm smaller than the canal diameter to facilitate smooth insertion.

Radial Fixation

For radial fixation, a 1-cm longitudinal incision was made on the lateral aspect of the distal radial metaphysis, either through the floor of the first dorsal compartment or between the second and third compartments near the Lister tubercle. Care was taken to avoid injury to the superficial branches of the radial nerve and extensor tendons. An entry hole was created using an awl, first perpendicularly and then obliquely toward the elbow, just proximal to the distal radial epiphysis. The fracture was reduced using external manipulation, and a pre-measured K-wire was advanced proximally through the fracture site with oscillating movements [2]. The wire was stopped short of the physis at the bicipital tuberosity level. Finally, the distal end of the K-wire was bent and cut 5-10 mm from the bone, and the skin was closed with one or two sutures.

Ulnar Fixation

For ulnar fixation, a stab incision was made over the lateral surface of the olecranon, approximately 2 cm distal to the physis. Under fluoroscopic guidance, an awl was used to create an entry hole into the ulna. The fracture was reduced, and a K-wire was advanced distally, stopping short of the physis. The wire was cut close to the bone, leaving enough length for easy removal, and the skin was closed. The ulnar nail was inserted through the tip of the olecranon or through the anconeus to avoid injury to the ulnar nerve, while the radial nail was inserted just proximal to the radial styloid or in the dorsal aspect of the distal radius proximal to the physis, avoiding the dorsal central start point to prevent injury to the extensor pollicis longus (EPL) tendon [12].

Postoperative care

An above-elbow splint was applied immediately after surgery. Patients were monitored overnight for pain, swelling, and neurovascular status. Intravenous antibiotics (ceftriaxone 50 mg/kg) were administered for 24 hours, followed by oral antibiotics (amoxicillin-clavulanate 20 mg/kg) for three days. The patients were discharged on the second postoperative day if they had adequate pain control, no signs of infection, and stable wound healing.

Follow-up

Patients were reviewed at one week for wound inspection and K-wire position assessment. The splint was removed after four weeks, and a below-elbow cast was applied for an additional two weeks. Radiographs were obtained at one, two, and three weeks postoperatively to monitor fracture alignment and healing. The cast was removed at six weeks if radiographs confirmed bridging callus formation and clinical examination revealed no tenderness or instability. Normal activities were permitted at this time, but sports were prohibited for three months. K-wires were removed between four and six months after surgery, depending on radiographic evidence of union. Physiotherapy was initiated at six weeks to restore the range of motion and strength.

Outcome measures

Functional outcomes were evaluated using the Price criteria [13], which assess symptoms and loss of forearm rotation. The Price criteria were selected because they provide a standardized and reproducible method for evaluating forearm function in pediatric patients. The outcomes were classified as excellent, good, fair, or poor based on the patient’s ability to perform strenuous work and the degree of rotational loss. The criteria are summarized in Table 1.

**Table 1:** Price criteria for clinical outcomes

Clinical outcome	Symptoms	Loss of forearm rotation
Excellent	No complaints with strenuous work	<11°
Good	Mild complaints with strenuous work	11°-30°
Fair	Mild complaints with daily work	31°-90°
Poor	All other results	>90°

Statistical analysis

Data were analyzed using SPSS Statistics for Windows, Version 16 (Released 2007; SPSS Inc., Chicago, United States). Descriptive statistics were used to summarize the demographic and clinical variables. Continuous variables are expressed as means and standard deviations, whereas categorical variables are presented as frequencies and percentages. Chi-square tests were used to compare categorical variables, and independent t-tests were used for continuous variables. A p-value of less than 0.05 was considered statistically significant.

## Results

Demographic characteristics

The study included 23 pediatric patients with unstable radius and ulna fractures treated with intramedullary K-wire fixation. The mean age of the patients was 10.4 years (range: 5-15 years). The majority of patients were male (69.6%, n=16), while females accounted for 30.4% (n=7). The distribution of cases according to age and sex is summarized in Table 2.

**Table 2:** Distribution of cases according to age and sex

Variable	Number of cases	Percentage
Age group		
5-10 years	11	47.8
11-15 years	12	52.2
Sex		
Male	16	69.6
Female	7	30.4

Mechanism of the injury

The most common mechanism of injury was road traffic accidents (RTAs), accounting for 52.2% (n=12) of cases, followed by falls (47.8%, n=11). The distribution of cases according to the injury mechanism is shown in Table 3.

**Table 3:** Distribution of cases according to the mechanism of injury

Mechanism of injury	Number of cases	Percentage
Road traffic accident (RTA)	12	52.2
Falls	11	47.8
Total	23	100

Fracture characteristics

The majority of fractures were transverse (56.5%, n=13), followed by oblique fractures (39.1%, n=9). Only one case (4.4%) involved a segmented fracture. The distribution of the fracture types is summarized in Table 4.

**Table 4:** Distribution of cases according to the fracture type

Fracture type	Number of cases	Percentage
Transverse	13	56.5
Oblique	9	39.1
Segmented	1	4.4
Total	23	100

Immobilization and fracture union

The average period of immobilization was 5.2 weeks (range: 4-6 weeks). Radiographic union was achieved in 82.6% (n=19) of cases within 12 weeks, whereas the remaining cases (17.4%, n=4) achieved union by 14 weeks. The distribution of cases according to the duration of immobilization and fracture union is shown in Table 5.

**Table 5:** Distribution of cases according to immobilization and fracture union

Duration	Number of cases	Percentage
Immobilization		
<4 weeks	4	17.4
4-6 weeks	14	60.9
>6 weeks	5	21.7
Fracture union		
<10 weeks	5	21.7
10-12 weeks	14	60.9
>12 weeks	4	17.4

Time of implant removal

The timing of K-wire removal varied among patients, with the majority (52.2%, n=12) undergoing removal between 12 and 14 weeks postoperatively. A smaller proportion (34.7%, n=8) had their implants removed after 14 weeks, whereas only 13.1% (n=3) had their implants removed before 12 weeks. The distribution of cases according to the time of implant removal is summarized in Table 6.

**Table 6:** Distribution of cases according to the implant removal time

Time of implant removal	Number of cases	Percentage
<12 weeks	3	13.1
12-14 weeks	12	52.2
>14 weeks	8	34.7
Total	23	100

Complications

Complications were observed in 34.8% (n=8) of cases, with pin site irritation being the most common (34.8%, n=8). Superficial wound infection occurred in 17.4% (n=4) of the cases, and it resolved with repeated dressings and a 10-day course of oral antibiotics. No deep infection, nonunion, or malunion cases were reported. The distribution of complications is summarized in Table 7.

**Table 7:** Distribution of cases according to complications

Complication	Number of cases	Percentage
Pin site irritation	8	34.8
Superficial infection	4	17.4
Total	12	52.2

Functional outcomes

According to the Price criteria, excellent results were achieved in 60.9% (n=14) of cases, whereas 30.4% (n=7) had good results. Fair results were observed in 8.7% (n=2) of cases, and no poor results were reported. The distribution of the functional outcomes is shown in Table 8.

**Table 8:** Distribution of cases according to the functional outcomes

Outcome	Symptoms	Loss of forearm rotation	Number of cases	Percentage
Excellent	No complaints with strenuous work	<11°	14	60.9
Good	Mild complaints with strenuous work	11°-30°	7	30.4
Fair	Mild complaints with daily work	31°-90°	2	8.7
Poor	All other results	>90°	0	0
Total			23	100

## Discussion

This prospective study evaluated the clinical outcomes of intramedullary K-wire fixation for unstable radius and ulna fractures in 23 pediatric patients. The results demonstrated that 60.9% (n=14) of patients achieved excellent functional outcomes and 30.4% (n=7) had good outcomes, according to the Price criteria. The complications were minimal, with 34.8% (n=8) of cases experiencing pin site irritation and 17.4% (n=4) developing superficial wound infections, all of which resolved with appropriate treatment. These findings suggest that K-wire fixation is a promising treatment option for unstable forearm fractures in children, particularly in resource-limited settings. However, the small sample size, single-center design, and short follow-up period necessitate cautious interpretation of the results.

Our results are consistent with recent studies that highlight the efficacy of intramedullary fixation for pediatric forearm fractures. For example, Varga et al. (2022) found that 89.4% of patients who were treated with intramedullary K-wires for distal forearm fractures had great outcomes, with a median union time of six weeks and few complications [14]. When researchers looked at antegrade elastic stable intramedullary nailing (a-ESIN) and transepiphyseal intramedullary K-wire fixation (TIK), they found that both methods gave stable fixation and full restoration of forearm function. The TIK group had no major complications [9].

When compared to other fixation methods, K-wire fixation offers several advantages. For example, it is minimally invasive, preserves the periosteal blood supply, and reduces the risk of soft tissue damage and scarring [15]. Additionally, the procedure is technically straightforward, requires a shorter operation time, and allows for early mobilization, which is particularly beneficial in pediatric patients [1]. In contrast, plate fixation provides better restoration of the radial bow but is associated with worse scar scores and higher costs [16]. ESIN offers perioperative advantages such as shorter operation time and reduced radiation exposure but is generally more expensive than K-wire fixation [17,18]. Both K-wire fixation and ESIN yield similar clinical outcomes, and the choice between them often depends on the surgeon’s experience and resource availability [19].

The timing of implant removal is an important consideration in pediatric fracture management because it reflects the healing process and influences patient outcomes. In our study, most patients (52.2%, n=12) had their K-wires removed between 12 and 14 weeks, which is consistent with the typical healing timeline for pediatric forearm fractures. This finding aligns with previous studies, such as Richter et al. (1998), who reported an average implant removal time of 12 weeks [20], and Chapman et al. (1989), who noted removal at 13 weeks [21].

The variation in removal time (13.1% before 12 weeks and 34.7% after 14 weeks) may reflect differences in fracture severity, patient age, and healing rates. For example, younger children tend to heal faster because of their higher remodeling potential, which may explain why some patients had their implants removed earlier. Conversely, delayed removal in some cases may have been due to slower healing or the need for additional stabilization.

Importantly, the timing of implant removal did not appear to significantly impact the functional outcomes in our study, as most patients achieved excellent or good results regardless of when the K-wires were removed. However, prolonged retention of implants may increase the risk of complications, such as pin site irritation and infection, as observed in 34.8% (n=8) of our cases. This highlights the need for careful monitoring and timely removal of implants to minimize complications and improve patient satisfaction.

Our study had a slightly higher complication rate (52.2%) compared to the recent literature. For example, a study on percutaneous K-wire fixation for distal radius fractures reported a complication rate of 37%, with pin site inflammation being the most common issue [10]. However, most complications in our study were minor (pin site irritation and superficial infections), and they resolved without long-term sequelae. This underscores the importance of meticulous postoperative care, including regular wound inspection and early intervention for pin site issues.

The use of intramedullary K-wires offers several advantages over other fixation methods, such as ORIF with plates. K-wire fixation is minimally invasive, preserves the periosteal blood supply, and reduces the risk of soft tissue damage and scarring [15]. Additionally, the procedure is technically straightforward, requires a shorter operation time, and allows for early mobilization, which is particularly beneficial in pediatric patients [1].

In our study, the average time to radiographic union was 10.4 weeks, which is comparable to the findings of recent studies. For example, a study by Abulsoud et al. (2023) reported a median union time of six weeks for distal forearm fractures treated with K-wires [1]. This further supports the efficacy of K-wire fixation in achieving timely fracture healing.

Strengths and limitations

This study has several notable strengths. First, it is a prospective study, which enhances the reliability of the data and reduces recall bias. Second, we used standardized outcome measures, such as the Price criteria, to evaluate functional recovery, ensuring consistency and reproducibility in our assessments. Third, the study was conducted in a real-world clinical setting, making the findings highly relevant to daily practice, particularly in resource-limited environments where K-wire fixation is a practical and cost-effective option. Additionally, our results align with previous studies, further validating the efficacy and safety of intramedullary K-wire fixation for pediatric forearm fractures.

However, the study also has several limitations. First, the small sample size (n=23) may limit the statistical power and generalizability of the findings. Second, the single-center design may introduce selection bias, as the patient population and surgical practices at our institution may not be representative of other settings. Third, the short follow-up period (six months) may not capture long-term outcomes, such as growth disturbances or late complications. Fourth, the study did not include a comparison group (e.g., patients treated with elastic stable intramedullary nailing (ESIN) or plate fixation), which limits our ability to draw conclusions about the relative efficacy of K-wire fixation. Finally, the statistical analysis was limited to descriptive statistics and basic comparisons; future studies could benefit from multivariate or subgroup analyses to identify factors associated with better outcomes.

Future studies should focus on long-term outcomes, such as growth disturbances and late complications, to fully evaluate the durability of K-wire fixation. Additionally, comparative studies evaluating K-wire fixation versus other surgical techniques, such as ESIN or plate fixation, would provide valuable insights into the optimal management of pediatric forearm fractures. For example, a study comparing bioresorbable implants with K-wires found that bioresorbable implants had fewer complications and did not require a second surgical intervention for removal, suggesting a potential alternative to traditional K-wire fixation [14]. Such research will help refine treatment guidelines and improve outcomes for children with these injuries.

## Conclusions

Intramedullary K-wire fixation is a promising treatment option for unstable radial and ulnar fractures in children, with favorable functional outcomes and manageable complications. The technique is particularly suitable for resource-limited settings due to its simplicity, cost-effectiveness, and minimal invasiveness. However, the small sample size, single-center design, and short follow-up period limit the strength of our conclusions. While our findings are encouraging, they should be interpreted as preliminary, and further research is needed to confirm the long-term efficacy and safety of this technique. Future studies should also explore comparative outcomes with other fixation methods to identify the optimal treatment approach for pediatric forearm fractures.
